# SIBLINGS’ EDUCATIONAL DIFFERENTIALS AND LATER-LIFE COGNITIVE HEALTH

**DOI:** 10.1093/geroni/igae098.1357

**Published:** 2024-12-31

**Authors:** Yue Qin, Michal Engelman

**Affiliations:** University of Wisconsin Madison, Madison, Wisconsin, United States; University of Wisconsin Madison, Madison, Wisconsin, United States

## Abstract

Drawing on data from 3,216 Wisconsin Longitudinal Study participants aged 79-84 years old with at least one sibling, we examined the relationship between siblings’ educational differentials and a unique set of late-life cognitive metrics. Results from OLS and multinomial logit regressions showed that higher educational attainment relative to one’s only sibling is associated with a higher Telephone Screening of Cognitive Status (TICS) score and marginally lower risks of cognitive impairment. For respondents with two or more siblings, however, siblings’ higher educational attainment relative to respondents was associated with a lower risk of cognitive impairment, and particularly a lower risk of Alzheimer’s Disease (AD), compared with individual higher educational attainment than siblings’ average education. Our findings suggest that both individual educational attainment and a better educated set of siblings can act as protective factors for later life cognition. Familial risk may matter more for AD than other types of impairment because of its genetic etiology, but further research should explore additional life-course, family resource, and network mechanisms that shape cognitive risk and resilience among and across siblings.

